# Television viewing and cognitive decline in older age: findings from the English Longitudinal Study of Ageing

**DOI:** 10.1038/s41598-019-39354-4

**Published:** 2019-02-28

**Authors:** Daisy Fancourt, Andrew Steptoe

**Affiliations:** Research Department of Behavioural Science and Health, University College London, London, UK

## Abstract

There has been significant interest in the effects of television on cognition in children, but much less research has been carried out into the effects in older adults. This study aimed to explore whether television viewing behaviours in adults aged 50 or over are associated with a decline in cognition. Using data from the English Longitudinal Study of Aging involving 3,662 adults aged 50+, we used multivariate linear regression models to explore longitudinal associations between baseline television watching (2008/2009) and cognition 6 years later (2014/2015) while controlling for demographic factors, socio-economic status, depression, physical health, health behaviours and a range of other sedentary behaviours. Watching television for more than 3.5 hours per day is associated with a dose-response decline in verbal memory over the following six years, independent of confounding variables. These results are found in particular amongst those with better cognition at baseline and are robust to a range of sensitivity analyses exploring reverse causality, differential non-response and stability of television viewing. Watching television is not longitudinally associated with changes in semantic fluency. Overall our results provide preliminary data to suggest that television viewing for more than 3.5 hours per day is related to cognitive decline.

## Introduction

Television has been described as a unique cultural activity in that it combines strong rapidly-changing fragmentary dense sensory stimuli on the one hand with passivity from the viewer on the other^1^. As a result of this unique combination, there has been interest for over a decade in the effect of television viewing behaviours on cognition. Much of this literature has concentrated on children. Despite some such studies showing positive associations with language acquisition and visual motor skills in very young children^2^, many more studies have shown concerning cognitive associations including with poorer reading recognition, reading comprehension and maths^3^, and cognitive, language and motor developmental delays^4,5^. However, much less attention has been paid to the effects of television viewing at the other end of the lifespan. Indeed, despite it having been hypothesised for over 25 years that watching excessive television can contribute to the development of dementia^1^, this theory still remains underexplored empirically. Additionally, there are no specific guidelines for recommended levels of viewing in older adults.

A few cross-sectional studies have suggested that present and past television viewing is associated with poorer cognition and an increased risk of developing Alzheimer’s disease^6–8^. But the small number of longitudinal studies that have been carried out have found mixed results. High levels of television viewing in young adulthood (>3 hours per day) through the following 25 years have been found to predict semantic fluency but not memory^9^ (although this study did not adjust for baseline cognitive function); >4 hours per day of television viewing amongst adults aged 37–73 has been longitudinally linked over 4 years with poorer short-term memory and fluid intelligence (but not clearly with visual-spatial memory)^10^; and television viewing in older adults has been associated with cognitive impairment up to 6 years later using the Mini Mental State Examination (MMSE)^11^. However, other studies have reported no association between watching television for more than 6 hours per day and either cognitive function two years later^12^, change in TV viewing patterns and cognition 6 years later^13^, or combined television and radio viewing/listening frequency and dementia onset over the following 6.4 years^14^.

An important feature to note is that many of these previous studies have looked at television not as the unique cultural activity described above but rather as a proxy for sedentary behaviour^9,12^. Sedentary behaviour has been independently linked with cognitive decline in older adults, including through vascular risk factors^15^. However, other sedentary activities such as using the internet are not associated with cognitive decline and might even contribute to cognitive preservation and reduced dementia risk^16^, which suggests that the sedentary nature of television watching does not solely explain its longitudinal associations with cognition.

Consequently, this study aimed to identify whether watching television in adults aged 50 or over is associated with decreased cognition six years later independent of sedentary behaviours in daily life. Further, most previous studies have focused on ‘excessive’ television watching (for example greater than 6 hours a day). But it remains unclear whether more moderate levels of television watching, carried out by a larger proportion of the population, might pose risks for cognition in older adults. So a secondary aim of this study was to identify what quantity of television can be viewed on a daily basis before associations with impairments in cognition are found.

## Results

We analysed data from the English Longitudinal Study of Aging (ELSA); a nationally-representative English longitudinal panel study of adults aged 50+^17^. Baseline data for this analysis were taken from Wave 4 of the study (2008–2009) and participants were followed up 6 years later at Wave 7 (2014–2015). We worked specifically with data from 3,590 participants who were free from dementia at baseline.

Participants had an average age of 67.1 years (SD = 7.7, range 52 to 90+). A total of 43.7% were male, 72.3% were married or cohabiting and 98.5% were white. Regarding employment, 14.8% reported working full time, 15.4% part time and the remainder no longer worked. We calculated the average number of hours of television watched per day, which we split into approximate quintiles: <2.5 hours per day (19.6%), 2.5–3.5 hours per day (19.1%), 3.5–4.5 hours per day (18.4%), 4.5–7 hours per day (23.4%), >7 hours per day (19.6%). Comparative analyses showed that women watched more television per day than men, as did people who were not married or cohabiting, people who were not working and those of a lower educational and wealth status (Table 1). While age varied between groups, this was only very slight (less than 22 month average difference).

**Table 1:** Differences in television viewing patterns dependent on key demographic factors.

	Total (N = 3,590)	<2.5 hrs/day	2.5–3.5 hrs/day	3.5–4.5 hrs/day	4.5–7 hrs/day	>7 hrs/day	Test statistic
Age (mean, SD)	67.1 (7.7)	66.1 (7.8)	66.7 (7.4)	67.4 (7.8)	68.0 (8.0)	67.1 (7.4)	**F**_**4**,**3585**_** = 6**.**63**
**Sex**							
Men	43.8%	23.8%	20.5%	16.8%	20.8%	18.0%	**Χ**^**2**^**(4) = 44**.**2**
Women	56.2%	16.2%	18.0%	19.6%	25.5%	20.7%	
**Marital status**							
Married/cohabiting	72.4%	21.1%	19.8%	18.1%	22.3%	18.8%	**Χ**^**2**^**(4) = 21**.**2**
Not married	27.6%	15.6%	17.4%	19.0%	26.5%	21.5%	
**Employment**							
Full time	14.9%	28.2%	23.6%	16.1%	16.5%	15.7%	**Χ**^**2**^**(8) = 80**.**5**
Part time	15.4%	24.2%	20.1%	19.9%	17.7%	18.1%	
Not working	69.7%	16.7%	18.0%	18.5%	26.2%	20.7%	
Retired	9.1%	17.9%	17.5%	20.9%	21.5%	22.2%	Χ^2^(4) = 4.06
**Education**							
No educational qualifications	27.5%	7.6%	12.7%	16.8%	32.5%	30.4%	**Χ**^**2**^**(3) = 399**.**1**^**a**^
Education qualifications at 16	19.5%	16.9%	19.6%	21.0%	23.6%	19.0%	
Education qualifications at 18	34.8%	19.1%	21.5%	19.3%	22.8%	17.3%	
Degree	18.2%	41.3%	23.9%	16.1%	10.7%	8.1%	
**Wealth**							
Lower quintile	19.4%	8.2%	11.3%	17.1%	32.9%	30.6%	**Χ**^**2**^**(4) = 323**.**3**^**a**^
2^nd^ quintile	20.0%	12.1%	17.1%	18.5%	29.1%	23.1%	
3^rd^ quintile	20.1%	17.0%	19.3%	21.1%	22.7%	19.9%	
4^th^ quintile	20.3%	27.6%	21.6%	18.5%	17.6%	14.7%	
Highest quintile	20.2%	32.3%	25.9%	16.6%	15.3%	9.9%	
**Chronic condition**							
New chronic condition	10.2%	21.6%	20.5%	16.7%	21.9%	19.4%	Χ^2^(4) = 2.3
No new chronic condition	89.8%	19.3%	19.0%	18.6%	23.6%	19.6%	
**Ongoing chronic condition**							
Ongoing chronic condition	3.0%	6.4%	11.9%	15.6%	35.8%	30.3%	**Χ**^**2**^**(4) = 27**.**3**
No ongoing chronic condition	97.0%	20.0%	19.3%	18.4%	23.0%	19.2%	
**Physical activity level**							
Sedentary/low	24.4%	12.5%	15.1%	17.5%	29.8%	25.2%	**Χ**^**2**^**(2) = 96**.**3**^**a**^
Moderately active	53.2%	19.1%	20.0%	19.0%	23.4%	18.4%	
Highly active	22.4%	28.2%	21.4%	17.8%	16.5%	16.2%	
Not leaving the house	45.3%	16.5%	18.1%	17.7%	26.7%	21.1%	**Χ**^**2**^**(4) = 34**.**5**
Mobility problem	7.9%	12.3%	9.8%	16.1%	31.9%	29.8%	**Χ**^**2**^**(4) = 49**.**4**
Reading a daily newspaper	62.8%	19.4%	18.5%	18.2%	24.1%	19.8%	Χ^2^(4) = 34.5
Using the internet	54.7%	26.3%	22.3%	17.7%	19.3%	14.3%	**Χ**^**2**^**(4) = 34**.**5**

^a^Kruskall-Wallis Test; boldface indicates statistical significance (*P* < 0.001).

Cognition was measured through frequently-used tests for verbal memory and semantic fluency (for cognition scores at baseline and follow-up, see Supplementary Table 1). The longitudinal associations between television viewing at baseline and cognition 6 years later was assessed using ordinary least squares regression, with the lowest level of television viewing (<2.5 hours of per day) as the reference category. Model 1 was adjusted for baseline cognition. Model 2 was additionally adjusted for demographic covariates (sex, age, marital status, ethnicity, educational attainment, employment status, retirement status, wealth and social support) and health-related covariates (depression, self-reported physical health, smoking, alcohol consumption and both long-standing and new chronic conditions including cardiovascular conditions). Model 3 was additionally adjusted for a range of sedentary behaviours (physical activity level, mobility problems preventing walking, not leaving the house, reading a daily newspaper and using the internet).

### Verbal memory

Watching television for more than 3.5 hours per day was associated with poorer verbal memory six years later with evidence of a dose-response relationship: greater hours of television per day were associated with poorer verbal memory at follow up (Table 2). While 44–55% of this association was accounted for by identified confounding variables, results were not attenuated, suggesting the finding existed independent of baseline cognition, demographic factors, health-related factors and sedentary behaviours. We tested the fully-adjusted model for trend, which provided further support for a linear relationship between television viewing and verbal memory (B = −0.13, SE = 0.04, *P* < 0.001, CI −0.20, −0.06). When comparing the size of this negative association with other predictors of cognitive decline, watching television for >3.5 hours a day had a greater sized negative association (standardised beta = −0.034) than being in the lowest wealth quintile (as compared with the median quintile: standardised beta −0.027), while watching television for >7 hours a day had a greater sized association (standardized beta = −0.048) as being in the highest wealth quintile (compared with the median quintile: standardised beta = 0.043) or having no educational qualifications (standardized beta = −0.058).

**Table 2:** Regression coefficients showing the longitudinal association between television and cognition.

	Verbal memory			Semantic fluency		
	B ± SE	p	CI	B ± SE	p	CI
**Model 1**						
<2.5 hrs/day	REF					
2.5–3.5 hrs/day	−0.09 ± 0.16	0.58	−0.41 to 0.23	−0.08 ± 0.31	0.80	−0.69 to 0.53
3.5–4.5 hrs/day	**−0**.**58 ± 0**.**16**	**<0**.**001**	**−0**.**90 to −0**.**26**	**−1**.**06 ± 0**.**32**	**<0**.**001**	**−1**.**68** to -**0**.**44**
4.5–7 hrs/day	**−0**.**86 ± 0**.**15**	**<0**.**001**	**−1**.**16 to −0**.**56**	**−1**.**21 ± 0**.**30**	**<0**.**001**	**−1**.**80 to −0**.**62**
>7 hrs/day	**−0**.**84 ± 0**.**16**	**<0**.**001**	**−1**.**16 to −0**.**53**	**−1**.**49 ± 0**.**31**	**<0**.**001**	**−2**.**10 to −0**.**87**
**Model 2**						
<2.5 hrs/day	REF					
2.5–3.5 hrs/day	0.01 ± 0.15	0.93	−0.29 to 0.31	0.15 ± 0.30	0.51	−0.44 to 0.75
3.5–4.5 hrs/day	**−0**.**34 ± 0**.**16**	**0**.**030**	**−0**.**64 to −0**.**03**	−0.51 ± 0.31	0.10	−1.12 to 0.10
4.5–7 hrs/day	**−0**.**39 ± 0**.**15**	**0**.**011**	**−0**.**69 to −0**.**09**	−0.22 ± 0.30	0.47	−0.82 to 0.38
>7 hrs/day	**−0**.**50 ± 0**.**16**	**0**.**002**	**−0**.**82 to −0**.**19**	−0.62 ± 0.32	0.051	−1.25 to 0.003
**Model 3**						
<2.5 hrs/day	REF					
2.5–3.5 hrs/day	0.001 ± 0.15	>0.99	−0.30 to 0.30	0.09 ± 0.30	0.77	−0.50 to 0.69
3.5–4.5 hrs/day	**−0**.**32 ± 0**.**16**	**0**.**040**	**−0**.**63 to −0**.**01**	−0.44 ± 0.31	0.16	−1.05 to 0.17
4.5–7 hrs/day	**−0**.**38 ± 0**.**15**	**0**.**014**	**−0**.**68 to −0**.**08**	−0.19 ± 0.31	0.53	−0.79 to 0.41
>7 hrs/day	**−0**.**45 ± 0**.**16**	**0**.**005**	**−0**.**77 to −0**.**14**	−0.50 ± 0.32	0.12	−1.13 to 0.13

Boldface indicates statistical significance; B = B coefficient; SE = standard error; CI = confidence intervals. Model 1 is adjusted for baseline cognition. Model 2 is additionally adjusted for demographic covariates (sex, age, marital status, ethnicity, educational attainment, employment status, retirement status, wealth, social support) and health-related covariates (depression, self-reported physical health, smoking, alcohol consumption and both long-standing and new chronic conditions). Model 3 is additionally adjusted for sedentary behaviours (physical activity level, mobility problems preventing walking, not leaving the house, reading a daily newspaper and using the internet). N = 3,590.

### Semantic fluency

Watching television for more than 3.5 hours per day was associated with poorer semantic fluency six years later in unadjusted models, but these results were attenuated by the inclusion of identified confounding demographic and health-related variables in the model. In particular demographic factors (age, ethnicity, lack of any educational qualifications and high wealth) and additionally both self-reported health and social engagement appear to have been key confounding variables leading to the attenuation of results. We tested the fully-adjusted model for trend, but it was unclear whether there was a linear relationship between television viewing and semantic fluency (B = −0.13, SE = 0.07, *P* = 0.082, CI −0.27, −0.02).

### Sensitivity analyses

In order to help guard against reverse causality, we excluded participants who reported a physician diagnosis of dementia in the two years following baseline, but this did not change the significance of results (Table S2). To manage attrition between baseline and follow-up, we applied inverse probability weighting using cross-sectional weights to correct for differential non-response, but this also did not materially affect results (Table S3). To ascertain whether certain groups of people showed clearer associations between television and cognition than others we respectively included age and baseline cognition as interaction terms with television viewing to assess whether they acted as moderators. These analyses suggested a moderation role of baseline verbal memory (B = −0.01, SE = 0.004, *P* = 0.002, CI 0.01, 0.02) but not age (B = −0.14, SE = 0.01, *P* = 0.18, CI −0.04, 0.01). Graphs of predictive margins showed that the associations were particularly marked amongst those with better verbal memory at baseline (Table S1). Fourth, we explored the stability of TV viewing behaviours over time. The average number of hours of TV viewing at baseline was strongly correlated with the number of hours of viewing 2 years later (r = 0.58, *P* < 0.001), attesting to a relative continuity of behaviours in older age. In order to explore this further, we computed an average viewing time across the 2 years and re-ran analyses, but this did not affect the significance of results (Table S4). Considering cardiovascular risk factors as well as pre-existing cardiovascular conditions (such as BMI and high blood pressure) did not attenuate results (Table S5). We also considered whether some of our identified covariates could in fact lie on the causal pathway, but a re-analysis with simplified covariate models provided comparable results (Table S6). Finally, by stratifying TV viewing further, we were able to clarify that 3–3.5 hours of television viewing is not itself associated with poorer cognition but viewing for 3.5 hours or more was. Therefore 3.5 hours rather than 3 hours appeared to be an important threshold (Table S7).

## Discussion

This study showed that watching television for more than 3.5 hours per day is associated with poorer verbal memory but not semantic fluency six years later, independent of demographic factors, physical activity, health-related factors or sedentary behaviours and even amongst those with no mobility problems or dementia diagnoses at or shortly following baseline. These associations were found in particular amongst those with better verbal memory at baseline and were strengthened by the clear dose-response pattern noted in results. These findings support previous associations shown between television watching and MMSE in a cohort of older adults in China^11^, but demonstrating more clearly the quantity of television watching associated with cognitive decline.

While demographic factors accounted for over 65% of the variation in television viewing and verbal memory, the significance of associations was not attenuated by the inclusion of these factors. Notably, associations between television viewing and verbal memory remained even when considering a range of variables relating to sedentary behaviours, suggesting that it is not just the sedentary nature of television watching that is responsible for its relationship with cognition. Consequently, a key question is why watching television has such associations with cognition. Laboratory experiments specifically exploring the effects of television viewing on cognition have shown that television leads to a more alert but less focused brain. Television involves fast-paced changes in images, sounds and action and, unlike other screen-based activities such as internet use and gaming, television is the most passive way of receiving such stimuli. So while studies have shown lower EEG posterior alpha band oscillations indicative of functional activation in the brain in response to television^18^, they have simultaneously found poor short and long-term memory following viewing^19^ and low brain-wave activity^20^. Indeed, it is of note that other screen-based activities such as video gaming and using the internet which involve more interaction can in fact have cognitive benefits, leading to higher levels of frontal and central EEG activity^21^ and enhanced visual-spatial skills, problem-solving skills and cognition in older age^16^, suggesting that it is this alert-passive interaction that is a key feature of the effects of television viewing.

Another potential mechanism by which television might be associated with verbal memory is stress. In addition to any potential cognitive stress created through the alert-passive interaction while watching television, the content of the programme itself can be stressful, such as through the depiction of graphic scenes, violence or the creation of suspense. Analyses of UK television from 2001–2013 (covering the country and much of the period of the data collection for the study reported here) have shown between 2.1 and 11.5 violent scenes per hour in UK soap operas, with 40% of these being categorised as moderate or strong violence^22^. It has even been proposed that the vividness of such experiences is greater than real-world experiences of events such as violence, conflict or disasters, as the drama is enhanced for entertainment purposes^23^. Chronic stress is known to lead to increased levels of glucocorticoids, which can have a direct effect on the hippocampus due to the presence of glucocorticoid receptors in that region of the brain. Consequently, stress has been shown to lead to atrophy of the hippocampus and impaired neurogenesis^24^, alongside impairments in cognition^25^.

Finally, excessive television could be linked with verbal memory through displacing other, cognitively beneficial activities such as playing board games, reading and engaging with cultural activities^11,26^. This theory implies that the relationship between television viewing and memory is not entirely down to television having negative effects per se, but rather television reducing the amount of time that people spend on more activities that could contribute to cognitive preservation. However, this remains to be explored further in future studies.

This study is not suggesting that watching television in older adulthood confers no benefits. Indeed, research with adults has suggested that TV dramas in comparison with TV documentaries can increase performance in tests of theory of mind, suggesting that television can enhance understanding of others^27^. Educational television can be an efficient way of learning when programmes are designed appropriately^28^. Television has also been shown to be a form of escapism from difficult life circumstances^29^. Further, research investing the effects of television viewing in the context of people’s daily lives has found that adults routinely report television as a means of relaxing^30^ (although this should be considered in relation to the potentially stress-inducing effects of television viewing discussed earlier). Nevertheless, this study suggests that watching substantial amounts of television is longitudinally associated with poorer verbal memory in older adults.

The strengths of this study include its use of a relatively large, nationally representative cohort of older adults including repeated measures of cognition across a six-year span and its findings of a dose-response pattern that is robust to a range of sensitivity analyses. Something we cannot rule out in this study is the possibility of reverse causality: lower cognition leads to greater levels of television watching. Indeed, there is an association between the amount of TV watched and the ‘need for cognition’ (the amount to which individuals are inclined towards engaging in activities that require thinking), with viewers who have a lower need for cognition feeling less pleasant when they have nothing to do so being more likely to escape the pressure to think by watching TV^29^. However, we controlled for baseline cognition in our analyses, and the finding from our sensitivity analyses that those with better cognition at baseline are most affected suggests that the associations are not solely due to poorer cognition leading to greater television habits. Additionally, our study did not find significant associations between television viewing and semantic fluency. The animal naming task we used to measure semantic fluency is well respected^31^. But as it is a task that contains many different aspects of executive function alongside semantic fluency (including cognitive flexibility, inhibitory control, speed of functioning and also verbal memory), it remains unclear whether television viewing might affect other components of executive function. This remains to be explored through further studies. Finally, in this study we attempted to differentiate between television as a sedentary behaviour vs television as a screen-based activity by controlling for a range of variables considered as proxies for sedentary behaviour. However, we are limited by the variables available within ELSA and there is no direct measure of sedentary behaviours in the dataset, so it is possible that some of the association between television and cognition is still due to its sedentary nature. Future studies may also like to consider further whether dietary factors such as high fat intake could be mediators between watching television and cognitive decline, and also whether changes in television viewing can lead to increases or delays in the rate of cognitive decline.

This study has presented data showing longitudinal associations between watching television and a decline in verbal memory amongst older adults. The findings presented here lead to several other research questions. For example, do different types of television content have different effects on cognitive decline? Given the associations with cognitive decline shown here, is television viewing specifically a risk factor for the onset of dementia? Further, does television continue to have harmful associations with cognition after the onset of dementia? It is of interest that, although television viewing remains high in care homes where residents have less autonomy or control over their own viewing, the desire to watch television naturally declines amongst people with dementia as soon as symptoms of the disease become evident^32^, suggesting that it becomes less rewarding as cognition gets worse. In light of this, there are bespoke television programmes for people with dementia being developed, dubbed ‘cognitively congruent programmes’^33^. These have been found to be more meaningful and entertaining for viewers than regular TV programmes, leading to longer viewing time. However, whether such programmes have any cognitive benefit or whether they in fact contribute to further cognitive decline remains unclear. So an exploration of the impact of television on broader cognitive decline and on the cognitive function of people with dementia remains to be undertaken. In the meantime, the findings reported here suggest that television viewing for more than 3.5 hours per day may be related to cognitive decline.

## Methods

### Participants

Participants were drawn from the English Longitudinal Study of Aging (ELSA); a nationally-representative English longitudinal panel study of adults aged 50+^17^. Baseline data for this analysis were taken from Wave 4 of the study (2008–2009) and participants were followed up at Wave 7 (2014–2015); a 6-year follow-up. We worked with the data from core respondents, meaning they were not living in an institution. For this analysis, carried out in 2017, participants were included if they provided data across all variables measured at baseline and follow-up and excluded if they were blind (n = 8) or had a physician diagnosis of dementia at baseline (n = 17). This left 3,590 participants. ELSA received ethical approval from the National Research Ethics Committee and all participants gave informed consent. All methods were performed in accordance with relevant guidelines and regulations.

### Measures

To measure daily average television watching (T), participants were asked on average how many hours of television they watched each weekday and each weekend day and T was computed as T = [(5*weekday) + (2*weekend)]/7. This produced a linear scale of number of hours viewed, which was then categorised into approximate quintiles: <2.5 hours per day (19.6%), 2.5–3.5 hours per day (19.1%), 3.5–4.5 hours per day (18.4%), 4.5–7 hours per day (23.4%), >7 hours per day (19.6%).

Cognition was measured through frequently-used tests for verbal memory and semantic fluency. Immediate and delayed verbal verbal memory were assessed using a word learning task in which participants were presented with 10 common words by a taped voice, one every 2 seconds^34^. Participants had to recall as many words as possible immediately and after a short delay during which they completed other cognitive tests. Following the protocol of previous studies, the results for immediate and delayed recall were then combined to give an overall verbal memory variable. For semantic fluency, we used an animal naming task, asking participants to think of as many words from a particular category (in this case animals) as possible in under 1 minute^29^. This task focuses on semantic fluency but also combines various aspects of broader executive function including cognitive flexibility, processing speed, inhibitory control and verbal fluency. These measures were chosen as both verbal memory and semantic fluency have been shown to be predictors of clinically significant cognitive decline^35^ (see Table S1 for precise figures).

Demographic covariates identified using directed acyclic graphs (DAGs) and included sex (men as the reference category), age (a continuous variable), marital status (married or cohabiting vs never married, divorced, separated or widowed), ethnicity (white or other), educational attainment (no qualifications, education up to the age of 16/GSCE qualifications, education up to the age of 18/A level qualifications, and education past the age of 18/degree), employment status (not working, working part time or working full time), retirement status (reported being retired), and wealth (split into quintiles, referring to total net non-pension assets; identified as a robust indicator of socioeconomic circumstances and standard of living in ELSA analyses)^36^. Several further health-related potentially confounding variables were also identified. Television viewing has been found to be associated with depression^37^, which is itself associated with cognitive dysfunction, including verbal memory impairment^38^. Consequently, a final covariate model additionally adjusted for depression using the 8-item Centre for Epidemiologic Studies Depression Scale (CES-D); a scale used extensively in a range of population cohorts^39^. We further included variables assessing self-reported physical health (on a scale from 1 = excellent to 5 = poor) and whether participants had a chronic or major health condition (including lung disease, arthritis, osteoporosis, cancer, Parkinson’s disease, blood disorder such as leukaemia or lymphoma, a stroke, high blood pressure, a heart attack, heart disease, diabetes or any other heart problem) newly diagnosed in the past 2 years, or a longer-term chronic condition. And we factored in behavioural variables pertaining to whether participants smoked (Yes/No) and how much alcohol they drank (an 8-point frequency scale ranging from not at all in the last 12 months to almost every day). Social support (frequency of contact with family, friends or children) was also included. Given the research cited above on television as a sedentary behaviour, five covariates were included that captured different elements of activity. Physical activity was measured through self-reported frequency of participation in vigorous, moderate and light exercise or activities and categorised as previously described into three summary groups^40^: inactive (no moderate or vigorous activity on a weekly basis); moderate activity at least once a week and vigorous activity at least once a week. Reported problems in walking 100 yards was also used to ascertain whether participants were more likely to be sedentary. And in wave 6 of ELSA, participants also recorded the number of minutes they spent walking outside the house on the day prior to their interview. Participants who recorded not leaving the house received a further categorisation of low activity. Reading a daily newspaper and using the internet/email were also included as covariates as they have previously been used as proxies for sedentary behaviour^12^.

### Statistical analysis

Baseline associations between television viewing and demographic variables were assessed using chi-squared tests for categorical variables and Kruskall-Wallis rank test for ordinal variables. The longitudinal association between television viewing and cognition was assessed using ordinary least squares regression, with the lowest level of television viewing (<2.5 hours of per day) as the reference category. Model 1 was adjusted for baseline cognition. Model 2 was additionally adjusted for demographic covariates (sex, age, marital status, ethnicity, educational attainment, employment status, retirement status, wealth and social support) and health-related covariates (depression, self-reported physical health, smoking, alcohol consumption and both long-standing and new chronic conditions). Model 3 was additionally adjusted for sedentary behaviours (physical activity level, mobility problems preventing walking, not leaving the house, reading a daily newspaper and using the internet). All models met regression assumptions.

Several sets of planned sensitivity analyses were also carried out. In order to guard against reverse causality, we excluded participants who reported a physician diagnosis of dementia in the two years following baseline. To manage attrition between baseline and follow-up, we applied inverse probability weighting using cross-sectional weights to correct for differential non-response. The third set of sensitivity analyses attempted to ascertain whether certain groups of people showed clearer associations between television and cognition than others. So they respectively included age and baseline cognition as interaction terms with television viewing to assess whether they acted as moderators. Our fourth set of sensitivity analyses explored the stability of TV viewing behaviours. The average number of hours of TV viewing at baseline (wave 4, 2008–2009) was strongly correlated with the number of hours of viewing 2 years later (wave 5, 2010–2011) (r = 0.58, *P* < 0.001), attesting to a relative continuity of behaviours in older age. But in order to explore this further, we computed an average viewing time across the 2 waves (wave 4 and wave 5) and re-ran analyses. This aimed to remove potential outliers who had reported unusually high or low levels of viewing at wave 4. Fifth, as well as taking account of cardiovascular conditions, we also considered whether cardiovascular risk factors could act as confounders in these analyses. So we additionally included high blood pressure (systolic BP ≥ 140 and diastolic BP ≥ 90) and BMI as categories (BMI < 25 underweight/normal, 25–30 pre-obese, or 30 + obese). Sixth, we were aware that our choice of covariates could potentially include factors that lie on the causal pathway. If this is the case then adjustment for them would not be appropriate. Therefore we also re-ran analyses with a simplified covariate model including only those covariates that could not lie on the causal pathway. Finally, we considered our thresholds of TV viewing. We re-ran our analyses using a further stratification to explore whether cut-offs of 3 or 3.5 hours per day might be more appropriate. The results of all sensitivity analyses are discussed and detailed tables of the results are given as supplementary material.

## Supplementary information

Supplementary Tables

## References

[1] Aronson M (1993). Does excessive television viewing contribute to the development of dementia?. Med. Hypotheses.

[2] Schmidt ME, Rich M, Rifas-Shiman SL, Oken E, Taveras EM (2009). Television Viewing in Infancy and Child Cognition at 3 Years of Age in a US Cohort. Pediatrics.

[3] Pagani LS, Fitzpatrick C, Barnett TA, Dubow E (2010). Prospective associations between early childhood television exposure and academic, psychosocial, and physical well-being by middle childhood. Arch. Pediatr. Adolesc. Med..

[4] Lin L-Y, Cherng R-J, Chen Y-J, Chen Y-J, Yang H-M (2015). Effects of television exposure on developmental skills among young children. Infant Behav. Dev..

[5] Zimmerman FJ, Christakis DA, Meltzoff AN (2007). Associations between media viewing and language development in children under age 2 years. J. Pediatr..

[6] Da Ronch C (2015). Association of television viewing with mental health and mild cognitive impairment in the elderly in three European countries, data from the MentDis_ICF65+project. Ment. Health Phys. Act..

[7] Fogel J, Carlson MC (2006). Soap operas and talk shows on television are associated with poorer cognition in older women. South. Med. J..

[8] Lindstrom HA (2005). The relationships between television viewing in midlife and the development of Alzheimer’s disease in a case-control study. Brain Cogn..

[9] Hoang, T. D. *et al*. Effect of Early Adult Patterns of Physical Activity and Television Viewing on Midlife Cognitive Function., Effect of early adult patterns of physical activity and television viewing on midlife cognitive function. *JAMA Psychiatry JAMA Psychiatry* **73**, 73, 73, 73–79 (2016). 10.1001/jamapsychiatry.2015.2468 PMC4755299 26629780

[10] Bakrania K (2018). Associations Between Sedentary Behaviors and Cognitive Function: Cross-Sectional and Prospective Findings From the UK Biobank. Am. J. Epidemiol..

[11] Wang JYJ (2006). Leisure activity and risk of cognitive impairment: The Chongqing aging study. Neurology.

[12] Hamer M, Stamatakis E (2014). Prospective Study of Sedentary Behavior, Risk of Depression, and Cognitive Impairment. Med. Sci. Sports Exerc..

[13] Kesse-Guyot E (2012). Cross-Sectional and Longitudinal Associations of Different Sedentary Behaviors with Cognitive Performance in Older Adults. Plos One.

[14] Wang H-X, Karp A, Winblad B, Fratiglioni L (2002). Late-life engagement in social and leisure activities is associated with a decreased risk of dementia: a longitudinal study from the Kungsholmen project. Am. J. Epidemiol..

[15] Wheeler MJ (2017). Sedentary behavior as a risk factor for cognitive decline? A focus on the influence of glycemic control in brain health. Alzheimers Dement. Transl. Res. Clin. Interv..

[16] d’Orsi, E. *et al*. Is use of the internet in midlife associated with lower dementia incidence? Results from the English Longitudinal Study of Ageing. *Aging Ment*. *Health* 1–9, 10.1080/13607863.2017.1360840 (2017). 10.1080/13607863.2017.1360840 PMC6127001 28795579

[17] Steptoe A, Breeze E, Banks J, Nazroo J (2013). Cohort Profile: The English Longitudinal Study of Ageing. Int. J. Epidemiol..

[18] Smith ME, Gevins A (2004). Attention and Brain Activity While Watching Television: Components of Viewer Engagement. Media Psychol..

[19] Uncapher MR, Thieu MK, Wagner AD (2016). Media multitasking and verbal memory: Differences in working verbal memory and long-term verbal memory. Psychon. Bull. Rev..

[20] Weinstein S, Appel V, Weinstein C (1980). Brain-activity responses to magazine and television advertising. J. Advert. Res..

[21] Pellouchoud E, Smith ME, McEvoy L, Gevins A (1999). Mental effort-related EEG modulation during video-game play: comparison between juvenile subjects with epilepsy and normal control subjects. Epilepsia.

[22] Cumberbatch, G., Lyne, V., Maguire, A. & Gauntlett, S. *Violence in UK soaps: a four wave trend analysis*. (Ofcom, 2014).

[23] Shrum, L. J. Media consumption and perceptions of social reality: effects and underlying processes. in Media Effects: Advances in Theory and Research (eds Bryant, J., Zillmann, D. & Oliver, M. B.) 69–96 (Routledge, 2002).

[24] Lupien SJ, Lepage M (2001). Stress, verbal memory, and the hippocampus: can’t live with it, can’t live without it. Behav. Brain Res..

[25] Squire LR (1992). Verbal memory and the hippocampus: a synthesis from findings with rats, monkeys, and humans. Psychol. Rev..

[26] Fancourt D, Steptoe A (2018). Cultural engagement predicts changes in cognitive function in older adults over a 10 year period: findings from the English Longitudinal Study of Ageing. Sci. Rep..

[27] Black, J. & L. Barnes, J. Fiction and Social Cognition: The Effect of Viewing Award-Winning Television Dramas on Theory of Mind. Psychol. Aesthet. Creat. Arts **9** (2015).

[28] Chu, G. & Schramm, W. *Learning from Television: What the Research Says*. (IAP, 2004).

[29] Henning B, Vorderer P (2001). Psychological escapism: predicting the amount of television viewing by need for cognition. J. Commun..

[30] Csikszentmihalyi M, Kubey R (1981). Television and the Rest ofLife: A Systematic Comparison of Subjective Experience. Public Opin. Q..

[31] Huppert FA, Cabelli ST, Matthews FE (2005). Brief cognitive assessment in a UK population sample – distributional properties and the relationship between the MMSE and an extended mental state examination. BMC Geriatr..

[32] Gústafsdóttir M (2015). Is Watching Television a Realistic Leisure Option for People withDementia. Dement. Geriatr. Cogn. Disord. Extra.

[33] Heller, R. B., Dobbs, B. M. & Strain, L. A. Video Programming for Individuals With Dementia: Assessing Cognitive Congruence. *American Journal of Alzheimer’s Disease & Other Dementiasr* **24**(2), 122–128 (2009). 10.1177/1533317508328051 PMC10846239 19075297

[34] Fillenbaum GG (2008). CERAD (Consortium to Establish a Registry for Alzheimer’s Disease) The first 20 years. Alzheimers Dement. J. Alzheimers Assoc..

[35] Masur DM, Sliwinski M, Lipton RB, Blau AD, Crystal HA (1994). Neuropsychological prediction of dementia and the absence of dementia in healthy elderly persons. Neurology.

[36] Banks, J., Karlsen, S. & Oldfield, Z. Socio-economic position. In *Health*, *Wealth and Lifestyles of the Older Population In England* (eds Marmot, M., Banks, J., Blundell, R., Lessof, C. & Nazroo, J.) 71–125 (2003).

[37] Hamer M, Stamatakis E, Mishra GD (2010). Television- and Screen-Based Activity and Mental Well-Being in Adults. Am. J. Prev. Med..

[38] Lam RW, Kennedy SH, McIntyre RS, Khullar A (2014). Cognitive Dysfunction in Major Depressive Disorder: Effects on Psychosocial Functioning and Implications for Treatment. Can. J. Psychiatry Rev. Can. Psychiatr..

[39] Steffick, D. E. *Documentation of affective functioning measures in the Health and Retirement Study*. (AnnARbor, MI: HRS Health Working Group, 2000).

[40] Hamer M, Lavoie KL, Bacon SL (2014). Taking up physical activity in later life and healthy ageing: the English longitudinal study of ageing. Br J Sports Med.

