# Supplementary material for: Television viewing and cognitive decline in older age: findings from the English Longitudinal Study of Ageing
Source: Sci Rep. 2019 Feb 28;9:2851. doi: 10.1038/s41598-019-39354-4 (PMC6395805; doi:10.1038/s41598-019-39354-4)
Supplement: Supplementary file 1 — Supplementary Tables [file 41598_2019_39354_MOESM1_ESM.docx]

Television viewing and cognitive decline in older age: findings from the English Longitudinal Study of Ageing

SUPPLEMENTARY MATERIAL

D Fancourt^a^* & A Steptoe^a^

^a^ Research Department of Behavioural Science and Health, University College London, UK

* Corresponding author: [d.fancourt@ucl.ac.uk](mailto:d.fancourt@ucl.ac.uk) 1-19 Torrington Place, London WC1E 7HB

**Table S1: Cognition scores at baseline and follow-up**

|  | Baseline mean (SD) | Follow-up mean (SD) |
| --- | --- | --- |
| Verbal memory | 10.95 (3.20) | 10.16 (3.71) |
| Executive function | 21.68 (6.54) | 20.76 (7.02) |

**Table S2: Regression coefficients showing the longitudinal association between television and cognition, omitting participants who went on, develop dementia in the two years following baseline**

|  | Verbal memory | | | Semantic fluency | | | |
| --- | --- | --- | --- | --- | --- | --- | --- |
|  | Beta (SE) | p | CI | Beta (SE) | | p | CI |
| <2.5 hrs/day | REF | | | |  |  |  |
| 2.5-3.5 hrs/day | -0.001 (0.15) | .99 | -0.30, 0.30 | 0.08 (0.30) | | .78 | -0.51, 0.68 |
| 3.5-4.5 hrs/day | **-0.32 (0.16)** | **.044** | **-0.63, -0.01** | -0.50 (0.31) | | .11 | -1.11, 0.11 |
| 4.5-7 hrs/day | **-0.39 (0.15)** | **.011** | **-0.70, -0.09** | -0.21 (0.31) | | .49 | -0.82, 0.39 |
| >7 hrs/day | **-0.47 (0.16)** | **.004** | **-0.79, -0.15** | -0.50 (0.32) | | .12 | -1.13, 0.13 |

Boldface indicates statistical significance; B=B coefficient; SE=standard error; CI=confidence intervals. Model adjusted for demographic covariates (sex, age, marital status, ethnicity, educational attainment, employment status, retirement status, wealth, social support) health-related covariates (depression, self-reported physical health, smoking, alcohol consumption and both long-standing and new chronic conditions) and sedentary behaviours (physical activity level, mobility problems preventing walking, not leaving the house, reading a daily newspaper and using the internet). N=3,472.

**Table S3: Regression coefficients showing the longitudinal association between television and cognition, weighted for attrition**

|  | Verbal memory | | | | Semantic fluency | | | | |  |
| --- | --- | --- | --- | --- | --- | --- | --- | --- | --- | --- |
|  | | Beta (SE) | p | CI | | Beta (SE) | | p | CI | |
| <2.5 hrs/day | | REF | | | | |  |  |  |  |
| 2.5-3.5 hrs/day | | 0.03 (0.16) | .84 | -0.35, 0.28 | | 0.004 (0.33) | | .99 | -0.65, 0.66 | |
| 3.5-4.5 hrs/day | | -0.30 (0.17) | .071 | -0.63, 0.03 | | -0.50 (0.33) | | .13 | -1.15, 0.15 | |
| 4.5-7 hrs/day | | **-0.39 (0.16)** | **.017** | **-0.71, -0.07** | | -0.28 (0.32) | | .39 | -0.91, 0.36 | |
| >7 hrs/day | | **-0.44 (0.17)** | **.009** | **-0.77, -0.11** | | -0.52 (0.33) | | .12 | -1.17, 0.13 | |

Boldface indicates statistical significance; B=B coefficient; SE=standard error; CI=confidence intervals. Model adjusted for demographic covariates (sex, age, marital status, ethnicity, educational attainment, employment status, retirement status, wealth, social support) health-related covariates (depression, self-reported physical health, smoking, alcohol consumption and both long-standing and new chronic conditions) and sedentary behaviours (physical activity level, mobility problems preventing walking, not leaving the house, reading a daily newspaper and using the internet). N=3,327.

**Table S4: Regression coefficients showing the longitudinal association between television and cognition, using an average of TV viewing behaviours at baseline and 2 years later**

|  | | Verbal memory | | | | Semantic fluency | | | | |
| --- | --- | --- | --- | --- | --- | --- | --- | --- | --- | --- |
|  | | Beta (SE) | | p | CI | Beta (SE) | | | p | CI |
| <2.5 hrs/day | | REF | | | | | |  |  |  |
| 2.5-3.5 hrs/day | | -0.33 (0.17) | | .05 | -0.65, -0.0003 | 0.17 (0.33) | | | .61 | -0.48, 0.82 |
| 3.5-4.5 hrs/day | | **-0.50 (0.17)** | | **.003** | **-0.84, -0.17** | -0.51 (0.34) | | | .13 | -1.17, 0.15 |
| 4.5-7 hrs/day | | **-0.55 (0.16)** | | **.001** | **-0.86, -0.23** | -0.25 (0.32) | | | .43 | -0.89, 0.38 |
| >7 hrs/day | | **-0.54 (0.17)** | | **.001** | **-0.87, -0.21** | -0.64 (0.33) | | | .057 | -1.29, 0.02 |

Boldface indicates statistical significance; B=B coefficient; SE=standard error; CI=confidence intervals. Model adjusted for demographic covariates (sex, age, marital status, ethnicity, educational attainment, employment status, retirement status, wealth, social support) health-related covariates (depression, self-reported physical health, smoking, alcohol consumption and both long-standing and new chronic conditions) and sedentary behaviours (physical activity level, mobility problems preventing walking, not leaving the house, reading a daily newspaper and using the internet). N=3,324.

**Table S5: Regression coefficients showing the longitudinal association between television and cognition, removing only adjusting for covariates that could not lie on the causal pathway**

|  | Verbal memory | | | Semantic fluency | | | |
| --- | --- | --- | --- | --- | --- | --- | --- |
|  | Beta (SE) | p | CI | Beta (SE) | | p | CI |
| <2.5 hrs/day | REF | | | |  |  |  |
| 2.5-3.5 hrs/day | 0.03 (0.16) | .86 | -0.29, 0.34 | 0.17 (0.32) | | .60 | -0.46, 0.80 |
| 3.5-4.5 hrs/day | **-0.27 (0.16)** | **.098** | **-0.59, 0.05** | -0.45 (0.32) | | .17 | -1.08, 0.19 |
| 4.5-7 hrs/day | **-0.34 (0.16)** | **.036** | **-0.66, -0.02** | -0.20 (0.32) | | .54 | -0.83, 0.44 |
| >7 hrs/day | **-0.42 (0.17)** | **.013** | **-0.75, -0.09** | -0.51 (0.34) | | .13 | -1.17, 0.15 |

Boldface indicates statistical significance; B=B coefficient; SE=standard error; CI=confidence intervals. Model adjusted for demographic covariates (sex, age, marital status, ethnicity, educational attainment, employment status, retirement status, wealth, social support) health-related covariates (depression, self-reported physical health, smoking, alcohol consumption and both long-standing and new chronic conditions, high blood pressure, BMI in categories) and sedentary behaviours (physical activity level, mobility problems preventing walking, not leaving the house, reading a daily newspaper and using the internet). N=3,189.

**Table S6: Regression coefficients showing the longitudinal association between television and cognition, removing only adjusting for covariates that could not lie on the causal pathway**

|  | Verbal memory | | | Semantic fluency | | | |
| --- | --- | --- | --- | --- | --- | --- | --- |
|  | Beta (SE) | p | CI | Beta (SE) | | p | CI |
| <2.5 hrs/day | REF | | | |  |  |  |
| 2.5-3.5 hrs/day | 0.02 (0.15) | .92 | -0.28, 0.31 | 0.17 (0.30) | | .57 | -0.42, 0.76 |
| 3.5-4.5 hrs/day | **-0.32 (0.16)** | **.04** | **-0.63, -0.01** | -0.47 (0.31) | | .13 | -1.08, 0.14 |
| 4.5-7 hrs/day | **-0.42 (0.15)** | **.006** | **-0.72, -0.12** | -0.28 (0.30) | | .36 | -0.88, 0.32 |
| >7 hrs/day | **-0.49 (0.16)** | **.002** | **-0.81, -0.18** | -0.61 (0.32) | | .056 | -1.24, 0.02 |

Boldface indicates statistical significance; B=B coefficient; SE=standard error; CI=confidence intervals. Model adjusted for demographic covariates (sex, age, marital status, ethnicity, educational attainment, employment status, retirement status, wealth, social support) health-related covariates (long-standing chronic conditions) and sedentary behaviours (mobility problems preventing walking, reading a daily newspaper and using the internet). N=3,590.

**Table S7: Regression coefficients showing the longitudinal association between television and cognition, using alternative thresholds of television viewing**

|  | Verbal memory | | | Semantic fluency | | | |
| --- | --- | --- | --- | --- | --- | --- | --- |
|  | Beta (SE) | p | CI | Beta (SE) | | p | CI |
|  |  | | | |  |  |  |
| <2.5 hrs/day | REF |  |  |  | |  |  |
| 2.5-3 hrs/day | -0.16 (0.17) | .37 | -0.49, 0.18 | -0.03 (0.34) | | .94 | -0.70, 0.65 |
| 3-3.5 hrs/day | -.27 (0.21) | .19 | -0.14, 0.69 | 0.30 (0.42) | | .47 | -0.52, 1.12 |
| 3.5-4.5 hrs/day | **-0.32 (0.16)** | **.04** | **-0.63, -0.02** | -0.44 (0.31) | | .16 | -1.05, 0.17 |
| 4.5-7 hrs/day | **-0.38 (0.15)** | **.014** | **-0.68, -0.08** | -0.19 (0.31) | | .53 | -0.79, 0.41 |
| >7 hrs/day | **-0.45 (0.16)** | **.005** | **-0.77, -0.14** | -0.50 (0.32) | | .12 | -1.13, 0.13 |

Boldface indicates statistical significance; B=B coefficient; SE=standard error; CI=confidence intervals. Model adjusted for demographic covariates (sex, age, marital status, ethnicity, educational attainment, employment status, retirement status, wealth, social support) health-related covariates (long-standing chronic conditions) and sedentary behaviours (mobility problems preventing walking, reading a daily newspaper and using the internet). N=3,590.
